# Microencapsulated Recombinant Human Epidermal Growth Factor Ameliorates Osteoarthritis in a Murine Model

**DOI:** 10.1155/2021/9163279

**Published:** 2021-09-23

**Authors:** Shih-Chao Lin, Xiang Zhang, Shiow-Yi Chen, Chi-Chien Lin, Yen-Shuo Chiu

**Affiliations:** ^1^Bachelor Degree Program in Marine Biotechnology, College of Life Sciences, National Taiwan Ocean University, Keelung 20224, Taiwan; ^2^Department of Physiology and Pharmacology, Karolinska Institute, Stockholm SE-171 77, Sweden; ^3^Department of Bioscience and Biotechnology, National Taiwan Ocean University, Keelung City 20224, Taiwan; ^4^Institute of Biomedical Sciences, National Chung Hsing University, Taichung 402, Taiwan; ^5^Department of Orthopedics, Shuang Ho Hospital, New Taipei 235, Taiwan; ^6^School of Nutrition and Health Sciences, College of Nutrition, Taipei Medical University, Taipei 110, Taiwan

## Abstract

Osteoarthritis, a highly age-related and chronic inflammatory disorder with cartilage loss, causes patients difficultly in movement; there is no efficient and sustainable remedy for osteoarthritis currently. Although hyaluronic acid (HA) and platelet-rich plasma (PRP) have been used to alleviate osteoarthritis, the effects could be short and multiple injections might be required. To address this issue, we exploited the property of chitosan to encapsulate recombinant human epidermal growth factor and obtained microencapsulated rhEGF (Me-rhEGF). In the current study, we induced the osteoarthritis-like symptoms with monosodium iodoacetate (MIA) in rats and investigated the therapeutic effects of Me-rhEGF. Following administration of HA/Me-rhEGF *in vivo*, we observed that the total Mankin scores, cartilage oligomeric protein, C-telopeptide of type II collagen, IL-1*β*, IL-6, IL-17A, and TNF-*α* cytokines, nitric oxide, and prostaglandin E2 expressions were significantly inhibited. Our results also strongly indicate that individual use of HA or rhEGF slightly decreased the inflammation and restored the destructive joint structure, but was not as drastic as seen in the HA/Me-rhEGF. Moreover, HA/Me-rhEGF profoundly reduced cartilage destruction and proteoglycan loss and downregulated matrix metalloproteinase expressions. These findings reveal that the treatment of HA/Me-rhEGF could be more beneficial than the use of single HA or rhEGF in reliving osteoarthritis and demonstrate the therapeutic application of microencapsulation technology in difficult joint disorders. In essence, we believe that the Me-rhEGF could be promising for further research and development as a clinical treatment against osteoarthritis.

## 1. Introduction

Osteoarthritis (OA) is a chronic, inflammatory, and degenerative joint disease, resulting in progressive cartilage damage. OA ultimately leads to cartilage loss, osteophyte formation (bone spurs), and bone thickening which in turn results in remodeling of affected joints. OA is more likely associated with patients that are elderly or obese. Estimably, there could be as high as 10–20% of people aged over 65 afflicted by OA [1]. At the age of 65, proteolytic enzyme levels increase in joint tissues, subsequently resulting in degradation of the structural proteins of cartilage. Examples of such structural proteins include type II collagen and cartilage oligomeric matrix protein (COMP). Excess body weight debilitates the strength of joints, exacerbating the inflammatory condition [2]. Patients with OA could present symptoms with pain and joint swelling in the spine, ankles, hips, or knees. The primary therapeutic approach to treat OA is almost exclusively through pain management via administration of nonsteroidal anti-inflammatory drugs (NSAIDs) or acetaminophen. However, such therapy does not successfully improve the symptoms or slow the progression of the disease [3].

In response to the lack of effective treatment, new remedies have been investigated for administration to OA patients, such as hyaluronic acid (HA). In 1997, the US Food and Drug Administration approved the use of HA by intra-articular injection to treat OA patients [4]. HA is one of the major components of synovial fluid. Additional supplement of HA can increase viscoelasticity and reduce the friction in joint tissues, attributing to the alleviation of inflammatory condition. However, the effect of HA injection may only persist for a number of weeks [5]. While a study in 1970 indicated that removing the HA from synovial fluid barely affected the joint lubrication [6]; visco-supplement injection might still be beneficial to OA patients with mild anti-inflammatory and placebo effects [7]. However, viscosity added by HA solution would eventually decrease to the level where lubricating property was lost, akin to the viscosity of water [8, 9].

Injection of growth factors, particularly platelet-rich plasma (PRP), has then been widely investigated as an alternative treatment of OA. However, due to the nonstandardized methods for PRP preparation and the various physiological conditions of individuals, such as comorbidities, age, and circulation, the effect of PRP is inconsistent and debatable. PRP contains multiple growth factors, including tissue growth factor-*β* (TGF-*β*), insulin-like growth factor-1 (IGF-1), platelet-derived growth factor (PDGF), vascular endothelial growth factor (VEGF), fibroblast growth factor (FGF), and hepatocyte growth factor (HGF) in addition to various cytokines, chemokines, and coagulation factors [10].

Among these growth factors, ligands of EGF receptor (EGFR) play an important role in protecting joint tissues from OA. EGF, as a ligand of EGFR, has been shown to be abundant in normal cartilage tissues [11]. However, direct and prompt injection of EGF to chronic inflammation in joint tissues could not be a feasible therapeutic approach due to the short half-life of EGF [12]. Therefore, a sustained and localized delivery of EGF in a spatial-temporally fashion is needed as a potential treatment of osteoarthritis.

Microencapsulation of a given drug with chitosan has been demonstrated to be a feasible delivery approach in nanomedicine. Chitosan could be obtained from chemical treatment of sodium hydroxide or enzymatic deacetylation [13, 14]. Chitosan-based delivery is particularly suitable for local administration, such as bronchitis or virginal mucosa as well as dermis [15]. Moreover, the chitosan-formed nanoparticles can be adjusted in size, ranging from 10–1000 nm [16, 17], and improve the bioavailability for water-insoluble natural products, such as flavonoids [18]. Here, we utilized polysaccharide-based chitosan to encapsulate rhEGF, supposed to prevent the degradation of rhEGF following administration *in vivo* and investigated the benefits of microencapsulation of rhEGF with HA as an excipient could be a novel approach in alleviation of OA. Ultimately, we attempted to demonstrate potential applications of bioactive components via this encapsulation technology.

## 2. Material and Methods

### 2.1. Preparation of Chitosan-Microencapsulated Recombinant Human Epidermal Growth Factor (Me-rhEGF)

Chitosan was generated from deacetylation of chitin followed by protonation of amino acid groups in an acidic environment [14]. rhEGF was added to the chitosan solution with pH 4.6–6, and then the solution was adjusted to pH 6–8. Protonated chitosan became water-soluble and easily interacted and stabilized with the negatively charged rhEGF to form particles (provided by GoodCare-Biotech, Ltd., Taipei, Taiwan, and patented by JOYCOM BIO-CHEM Co., Ltd.) [19, 20].

### 2.2. Animals

Six-week-old male Sprague-Dawley rats, weighted from 200 to 300 g, were purchased from the National Laboratory Animal Center (Taipei, Taiwan) and maintained in the National Chung Hsing University (NCHU) Animal Experiment Research Center following the guide for the care and use of laboratory animals. These animals were provided with sterilized and unlimited chow and water and the experimental protocol was approved by the IACUC of NCHU (Approval no. 107-011).

### 2.3. Induction of OA and Treatments

**Figure 1:**
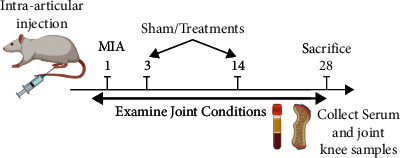
Schematic illustration of experimental design.

Monosodium iodoacetate (Sigma, St. Louis, MO, USA) was used to induce OA as described previously [21]. In brief, 1.5 mg of monosodium iodoacetate was dissolved in 30 *μ*L of 0.9% saline followed by a single intra-articular injection into the right knee joint per mouse; control mice were given a single injection of 0.9% saline. Treatments, HA, rhEGF, or HA/Me-rhEGF were also given to each group of rats (*n* = 5) on day 3 and 14 of the experiment duration via intra-articular administration within two weeks after injection of monosodium iodoacetate. Physiological and pathological changes were examined and determined by 4^th^ week after injection of monosodium iodoacetate (Figure 1).

### 2.4. Pathological and Histological Analyses of Joint Tissues

The sizes and microstructure of joint tissues were measured or examined with a calibrated digital caliper (Mitutoyo, Kawasaki, Japan) and hematoxylin and eosin (H&E) staining, respectively. Briefly, tissue sections were prepared following the sacrifice on the 4^th^ week. 5 micron sections of paraffin-impregnated tissues were subjected to H&E staining or safranin O-fast green staining (Sigma, St. Louis, MO) followed by examination of tissue structure and graded using a modified Mankin scoring system [22]. The final scores correlated with higher joint tissue destruction.

### 2.5. Analyses for Serum Biomarkers

Rat blood was collected at 4 weeks after rats were sacrificed. Rat blood samples were centrifugated at 1500 × *g* for 10 min to obtain serum. The concentrations of nitric oxide (NO) were determined by Griess assay to quantify the oxidation product of NO, nitrite [23]. Briefly, 100 *μ*M of sodium nitrate and Griess reagent (equal volumes of 2% sulfanilamide and 0.2% N-(1-naphthyl)ethylenediamine dihydrochloride) were added to a well in a 1 : 1 ratio followed by incubation for 10 min at room temperature and measurement at 540 nm by an ELISA reader.

Prostaglandin E2 (PGE2; Cusabio, Biotech, Wuhan, Hubei, China), proinflammatory cytokines, including IL-1*β*, IL-6, IL-17A, and TNF-*α* (Thermo Fisher Scientific, Waltham, MA, USA), and cartilage degeneration mediators, cartilage oligomeric matrix protein (COMP) (Cusabio), and c-terminal cross-linked telopeptides of type II collagen (CTX-II) (Lifespan Biosciences Corporations, Seattle, WA, USA) were determined by indicated ELISA kits. 100 *μ*L of diluted serum (50 *μ*L/well for PGE2 kit) was added to wells coated with capture antibodies and blocked with 5% BSA blocking buffer for 2 hrs followed by washing with PBS-T buffer (0.5% Tween-20 in PBS), 100 *μ*L/well biotinylated detection antibody for 1 h, and 100 *μ*L/well Avidin-HRP for 30 min at room temperature. Finally, 100 *μ*L/well TMB substrate was used to develop the colorimetric reaction which lastly measured by an ELISA reader at 450 nm after the reactions were terminated by ELISA stop solution (100 *μ*L/well of 0.16 M sulfuric acid).

### 2.6. Western Blot Analysis

The homogenates of rat cartilages were centrifuged twice at 12000 × *g* for 15 min at 4°C to isolate supernatant followed by quantification using a BCA Protein Assay Kit (Bio-Rad, Hercules, CA) and western blotting as described previously [24]. In brief, protein lysates were separated by SDS-PAGE and transferred to polyvinylidene fluoride (PVDF) membranes. The membranes were incubated with a blocking buffer (5% nonfat milk in Tris-buffered saline and Tween-20) followed by incubating with the following antibodies at 1 : 1000 dilutions: iNOS, COX-2, MMP-3, MMP-7, and GAPDH (all from Abcam, Cambridge, MA, USA) for overnight at 4°C. A 1 : 1000 dilution of goat-anti-rat IgG-conjugated with horseradish peroxidase (HRP; cat. no. 111-35-114; Jackson ImmunoResearch Laboratories, West Grove, PA, USA) secondary antibody was added to membranes for a 1-hour incubation. Protein detection was performed using an ECL Western Blotting Substrate Kit (ECL Prime Western Blotting Substrate; GE Healthcare Life Sciences, Piscataway, NJ, USA) and by imaging using the Hansor Luminescence Image System (Taichung, Taiwan). All the densitometric analysis for western blotting results was conducted using ImageJ v1.47 (Bethesda, MD, USA).

### 2.7. Prediction of Protein-Protein Interaction

Osteoarthritis-related proteins (DOID: 8398) were extracted from Human Disease Ontology database (https://disease-ontology.org/) via R package “Dose.” Protein-protein interactions network was constructed by R package “STRINGdb” and “Cytoscape” with interaction score = 0.9.

### 2.8. Statistics

All data were plotted as mean ± SEM unless stated elsewhere. The statistical significance between groups was defined as *P* value <0.05 using the student's *t*-test or one-way ANOVA with subsequent Tukey's HSD test with Prism v8.4 (GraphPad Inc., La Jolla, CA, USA).

## 3. Results and Discussion

**Figure 2:**
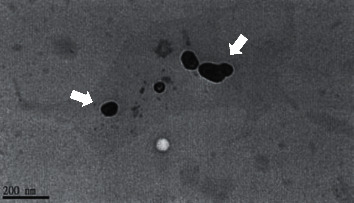
Image of chitosan-microencapsulated rhEGF from transmission electronic microscope (TEM).

By applying the microencapsulation technology mentioned earlier, we obtained the microencapsulated rhEGF for the entire study. Before ensuring the integrity structure of rhEGF, we utilized transmission electron microscope (TEM) to visualize the microencapsulated rhEGF. As shown in Figure 2, these microspheres were well-preserved in the polysaccharide (chitosan) shell with the sizes around dozens of nanometers, ranging from 50–200 nm.

**Figure 3:**
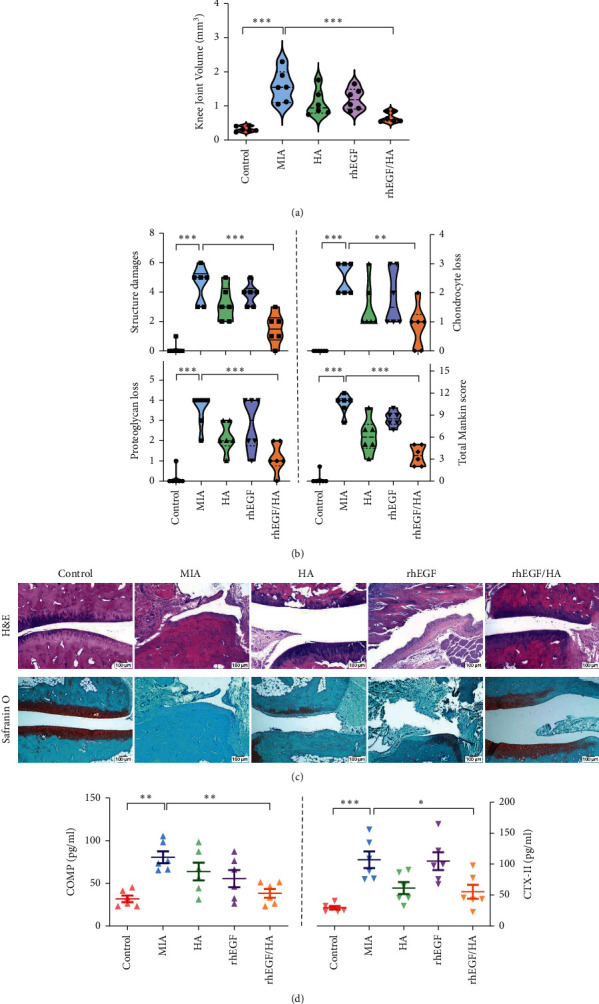
Effects of HA, rhEGF, and HA/Me-rhEGF on MIA-induced OA rats. (a) Sizes of knee joints measured by a knee diameter, and (b) joint lesions, including destructive tissue structure, chondrocyte and cartilage loss, and total Mankin grades, were examined and are presented as mean ± SEM. (c) Representative images of histological alterations in the knee joints as indicated by H&E staining and safranin O/fast green staining. (d) COMP and CTX-II in serum were measured by ELISA as cartilaginous degradation indicators (mean ± SEM from three independent experiments).

We then prompted to determine whether HA, rhEGF, or HA/Me-rhEGF could elicit any protective effects to ameliorate osteoarthritic symptoms in an animal model; we utilized monosodium iodoacetate (MIA), a selective inhibitor of glyceraldehyde-3-phosphate dehydrogenase, to induce osteoarthritis-like symptoms on rats. On 14 days of administration of MIA, animals exhibited swollen knees (Figure 3(a); MIA group). This was characterized with the pathological changes in the tissue microstructures, including clefts and irregular surfaces, a loss of proteoglycan (safranin-O staining) and cartilage, and elevated total Mankin scores (Figures 3(b) and 3(c); MIA group). Individual use of HA or rhEGF reduced the severity of OA in rats, but not significantly. The administration of HA/Me-rhEGF, in contrast, resulted in drastic decreases of knee swelling and stymied overall OA symptoms (Figures 3(a)–3(c); HA/Me-rhEGF group), demonstrating the protective effects of combined treatment with HA/Me-rhEGF.

**Figure 4:**
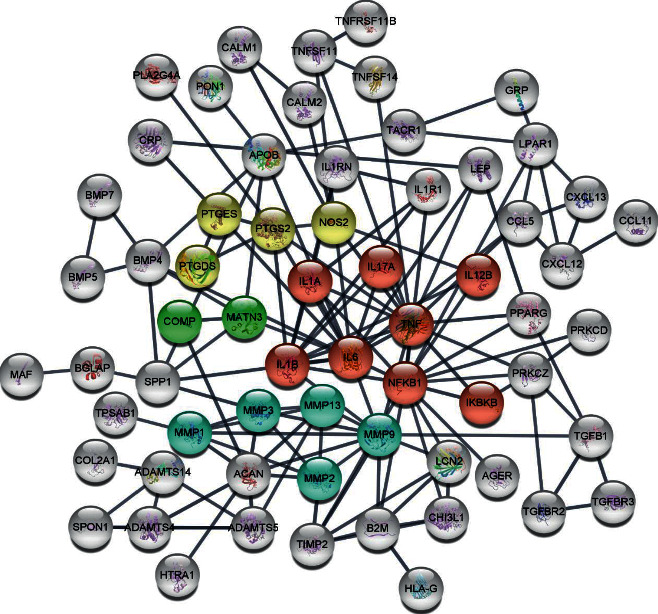
Prediction of osteoarthritis-related protein-protein interaction. The proteins were extracted from Human Disease Ontology database and the network was constructed by R packages. Color red designates inflammatory responses; yellow indicates the inflammatory mediators; green means osteoarthritis indication-related proteins; and blue means MMP cascade.

Levels of cartilage oligomeric protein (COMP) and C-telopeptide of type II collagen (CTX-II) in circulation are highly associated with the cartilage degradation and pathogenesis of OA [25]. Keeping this in mind, we quantified the concentrations of COMP and CTX-II in rat serum by ELISA. Levels of COMP among groups corresponded to the severity seen in histological evidences. On the other hand, the circulating level of CTX-II in rhEGF treatment group was barely inhibited and was not suppressed as effectively as seen in groups treated with only HA or HA/Me-rhEGF (Figure 3(d)). To further pin down the downstream protein expressions which could be related to osteoarthritis and prominently influenced by our designated treatments, HA, rhEGF, or HA/Me-rhEGF, we constructed the protein-protein interaction network following extraction of osteoarthritis-related proteins from Human Disease Ontology database. As highlighted in Figure 4, the inflammatory mediators, cytokines, and matrix metalloproteinases appeared to be worthwhile for further examinations.

**Figure 5:**
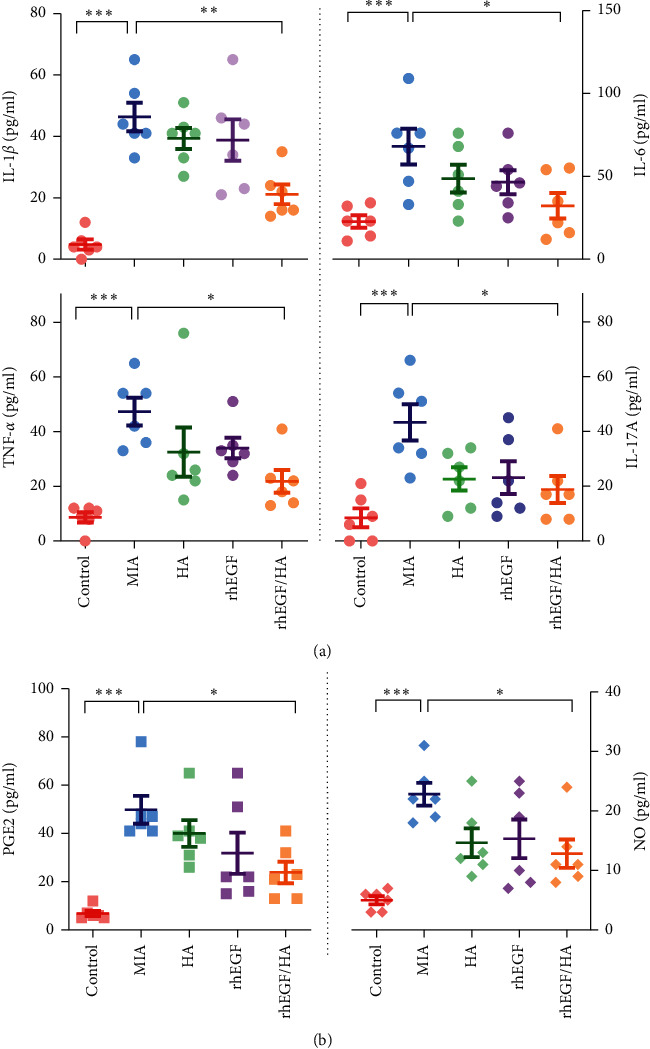
Inflammatory cytokines in circulating serum. The levels of (a) IL-1*β*, IL-6, TNF-*α,* and IL-17A and (b) inflammatory mediators, prostaglandin E2 (PGE2), and nitric oxide (NO) in serum samples were measured by ELISA or Griess reaction (NO) with or without treatments of HA, rhEGF, or HA/Me-rhEGF. Data are expressed as mean ± SEM from the three independent experiments.

As such, we investigated whether osteoarthritic inflammation was also alleviated by the treatments by examining the proinflammatory mediators and cytokines, such as prostaglandin E2 (PGE2), nitric oxide (NO), IL-1*β*, IL-6, TNF-*α,* and IL-17A. The data shown in Figure 5(a) were consistent with the histopathological changes shown in Figure 3 where the treatment of HA/Me-rhEGF reduced the circulating levels of proinflammatory mediators significantly. Despite the similar trends, the treatment of HA or rhEGF did not significantly result in lowered levels of proinflammatory cytokines, as was seen in combinational treatment.

**Figure 6:**
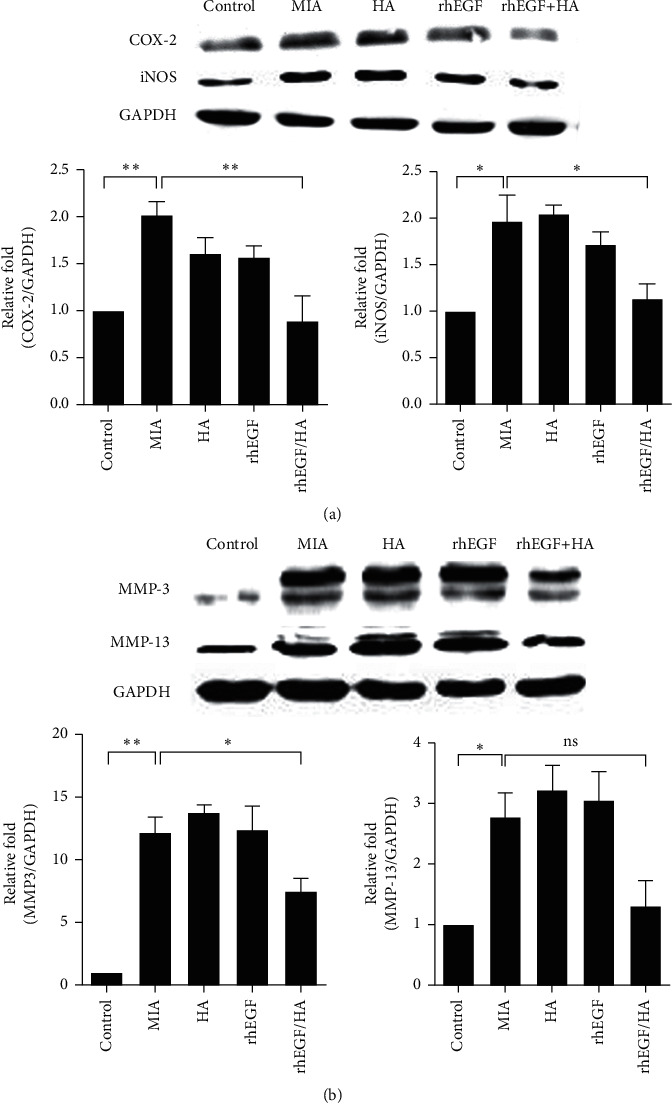
Effect of treatments on inflammatory mediators and MMPs in cartilaginous tissues. The expressional levels of (a) iNOS and COX-2 and (b) MMP-3 and MMP-13 were determined by western blot analyses in joint homogenates. GAPDH was used as a loading control, and protein expressions were quantified by ImageJ software and normalized based on GAPDH expression. Data are expressed as mean ± SEM from the three independent experiments.

Next, we evaluated the inflammatory mediators, particularly nitric oxide (NO) and prostaglandin E2 (PGE2) in serum and the changes of corresponding upstream signaling pathways via ELISA and western blot, respectively. Our data indicated that concentrations of NO and PGE2 in osteoarthritic rats were remarkably increased. In comparison, the individually administered HA or rhEGF specimens displayed a slight reduction of inflammatory mediators, and HA/Me-rhEGF treatment still showed the most significant reduction of aforementioned inflammatory mediators (Figure 5(b)). Moreover, the expressions of inducible nitric oxide synthase (iNOS) and cyclooxygenase-2 (COX-2) responsible for generation of NO and PGE2, respectively, were also downregulated in the cartilaginous tissues (Figure 6(a)). These data were consistent with the data obtained from ELISA. Our results suggest that HA/Me-rhEGF was efficiently able to reduce the inflammation in osteoarthritic rats.

Finally, we attempted to examine the protein levels of matrix metalloproteinase (MMPs) which could be closely associated with the pathogenesis of OA and some of them are known as therapeutic targets for OA [26, 27]. As our results show, the expression of MMP-3 and MMP-13 in OA rats was profoundly upregulated and, surprisingly, the administration of HA or rhEGF independently did not diminish levels of MMP-3 and MMP-13. However, the treatment of HA/Me-rhEGF reduced the level of MMP-3 remarkably as well as MMP-13 to some extent despite not significantly (Figure 6(b)), suggesting the administration of HA/Me-rhEGF could efficiently alleviate the MIA-induced OA through inhibition of MMP cascade.

Overall, our results indicate that direct administration of HA or rhEGF via intra-articular injection could partially ameliorate the pathological indexes of osteoarthritis induced with MIA, such as inflammatory mediators, NO and PGE2, and proinflammatory cytokines, but a significant effect was achieved upon treating with HA and chitosan-microencapsulated rhEGF. In addition to the combinational efficacy, we demonstrated that the two dosages of HA/Me-rhEGF prolonged the therapeutic duration in the MIA-induced OA model (Figure 3) compared to previous studies where at least three times of HA injections were required to observe the symptomatic alleviation in OA rats [28, 29], signifying the possibility to reduce the revisiting frequency for OA patients.

Despite the benefits of HA administration in OA, we observed that the HA combined with rhEGF were far more effective to stymie the arthritic symptoms. CD44 was shown to be the receptor of HA [30] and the expression level of CD44 on the articular cartilage was also highly associated with the severity of OA [31]; the binding of CD44 and HA inhibited the release of interleukin-1*β* and MMPs, including MMP-3 and 13 (Figures 5(a) and 6(b)) [32]. Despite potential benefits of HA, it is noted that HA alone is not able to substantially improve the lubricating condition in a joint cavity [6, 7, 9]. The lubrication effect mainly comes from the aggregation of glycoprotein in the extracellular matrix (ECM) [33], where EGF serves as a core protein to facilitate the glycoprotein aggregation in the joint cavity. In fact, such glycoprotein aggregations require the interaction between HA and EGF, which could further be cross-linked with fibulins in ECM and ultimately form protein-glycosaminoglycan complexes to provide necessary lubrication and harmonious physical status in joint tissues [34, 35]. Hence, HA and rhEGF could synergistically facilitate the improvement of OA conditions.

In conclusion, OA is a chronic inflammatory disease that is difficult to be completely treated. However, our study provides insight and promising results for the prompt delivery of HA/Me-rhEGF. Our novel therapeutic approach ensures a steady and consistent release of rhEGF coordinating with HA to ameliorate the symptoms of MIA-induced OA in rats. That is evidenced by significantly reduced inflammatory cytokines and mediators. Overall, chitosan-microencapsulated rhEGF exhibited higher stability in preservation and provided better anti-inflammatory effect than nonencapsulated rhEGF. As a result, we believe that this HA/Me-rhEGF has promising potential as an OA treatment in the clinical field.

## Data Availability

The data used to support the findings of this study are included within the article.
